# What factors influence women's empowerment in Ethiopia? A multilevel analysis of Ethiopia's demographic and health survey data

**DOI:** 10.3389/fgwh.2024.1463157

**Published:** 2024-10-31

**Authors:** Endalkachew Dellie, Misganaw Guadie Tiruneh, Melak Jejaw, Kaleb Assegid Demissie, Mihret Getnet, Tadele Biresaw Belachew, Getachew Teshale, Banchlay Addis, Demiss Mulatu Geberu, Lake Yazachew, Tesfahun Zemene Tafere, Nigusu Worku

**Affiliations:** ^1^ Department of Health Systems and Policy, Institute of Public Health, College of Medicine and Health Sciences, University of Gondar, Gondar, Ethiopia; ^2^ Department of Epidemiology and Biostatics, Institute of Public Health, University of Gondar, Gondar, Ethiopia

**Keywords:** justified wife-beating, multilevel analysis, Ethiopia, decision making, women's empowerment

## Abstract

**Background:**

Women's empowerment has been a global priority, as countries can achieve significant growth and economic development by empowering women. Understanding the individual and community-level factors that influence women's empowerment is crucial for policymakers to develop effective policies and to improve women's empowerment.

**Method:**

A community-based cross-sectional survey was conducted in 11 administrative regions of Ethiopia. The analysis included a weighted sample of 7,108 married women of reproductive age (15–49 years) from the 2,016 Ethiopian Demographic and Health Survey (EDHS). A multilevel mixed-effect binary logistic regression analysis was used to examine the individual and community-level factors associated with women's empowerment. In the final model, significant variables were identified using a *p*-value of <0.05 and an adjusted odds ratio (AOR) with a 95% confidence interval (CI).

**Results:**

The overall magnitude of women's empowerment was 23.7% (95% CI: 22.7–24.7). Only 30.9% of women reported participating in household decision-making, and 32.5% disagreed with all the reasons justifying wife-beating. At individual-level, factors positively associated with women's empowerment included secondary (AOR: 2.72 (1.77–4.23), and higher (AOR: 3.65 (1.81–7.34) education. However, belonging to the Muslim religion was negatively associated with women's empowerment (AOR: 0.63 (0.47–0.85). At the community level, wealthy communities were positively associated with women's empowerment (AOR: 1.60 (1.05–2.44). Conversely, residing in rural areas (AOR: 0.49 (0.29–0.83), and living in the Afar (AOR: 0.35 (0.17–0.70), Amhara (AOR: 0.45 (0.26–0.79), Oromia (AOR: 0.43 (0.26–0.73), South Nation Nationalities, and Peoples (SNNP) (AOR: 0.42 (0.24–0.75), and Gambella (AOR: 0.36 (0.20–0.66) regional states were negatively associated with women's empowerment.

**Conclusion:**

The overall magnitude of women's empowerment in this study was low. Factors that positively influenced empowerment included attending secondary and higher education, as well as residing in communities with higher wealth status. On the other hand, being Muslim, residing in rural areas, and living in the Afar, Amhara, Oromia, SNNPR, Gambella, and Tigray regions were negatively associated with women's empowerment. As a result, the government of Ethiopia needs to design community-based women's empowerment strategies and involve women in income-generation activities that improve their participation in household decision-making to empower them.

## Introduction

Women's empowerment is a fundamental driver of sustainable development and is a global priority agenda, especially in developing countries where gender disparities are often pronounced (1). It was one of the Millennium Development Goals (MDGs) and remains central to achieving the Sustainable Development Goals (SDGs (2, 3). Despite numerous efforts to promote gender equality, many women still face significant barriers to accessing education, economic opportunities, and healthcare services (4–7).

Women's empowerment is relatively a complex and multidimensional concept that centers on enhancing women's capacity to make strategic life choices, especially in situations where such opportunities have been denied to them (8, 9). Due to its multifaceted nature, objectively measuring women's empowerment poses challenges for researchers. This study assessed women's empowerment using index-based data from the nationally representative 2016 Ethiopian Demographic and Health Survey (EDHS), in terms of decision-making power and attitudes toward the justification of wife-beating.

Women's empowerment is a significant component of the development process. Economically independent and educated women are more likely to seek and utilize healthcare services, leading to better health outcomes for themselves and their families (5). Research conducted in 67 developing countries has shown a positive association between women's empowerment and the use of health services (10). For example, women with higher levels of empowerment are more likely to use reproductive health services, such as family planning, antenatal care, and skilled attended delivery (11, 12). Therefore, it is clear that women's empowerment is essential for promoting the well-being of households and children. Additionally, various studies worldwide have identified several factors contributing to women's empowerment, including women's education, age, media access, employment, place of residence, and income level (13–17).

The Ethiopian government has implemented various institutional and policy measures to promote gender equality and empower women. These efforts include the 1993 Ethiopian Constitution (18), the Ethiopian National Policy on Women (19), and the Growth and Transformation Plan (GTP) I and II (20, 21). Despite these measures, gaps in women's empowerment persist. For instance, a significant proportion of women still lack decision-making power in key areas; such as household purchases (21%), healthcare decisions (18%), and visits to their family or relatives (16%). Additionally, a considerable number of women (63%) disapprove of wife-beating for any reason (22). These evidences highlight the persistent challenges in achieving true gender equality in Ethiopia.

Furthermore, a study based on data from the Ethiopian Demographic and Health Survey (EDHS) conducted in 2011 found that about half of the women surveyed disagreed with wife-beating (23). However, this study only measured women's attitudes towards wife-beating and did not thoroughly analyze the individual and community-level factors influencing women's empowerment. Moreover, the previous studies used binary logistic regression, which did not comprehensively analyze both community-level and individual-level factors, potentially overlooking the issue of non-independence.

Understanding individual and community-level factors that are likely to influence women's empowerment may be a commencement in exploring strategies to empower women and cut back on gender inequality. Therefore, this study tried to examine the magnitude of women's empowerment and identify its determinants using a multilevel model, from the 2016 EDHS data.

## Methods

### Study settings and period

Ethiopia is located in the Horn of Africa and shares borders with Eritrea, Djibouti, Somalia, Sudan, South Sudan, and Kenya. It is the second most populous nation on the African continent after Nigeria, with a population of 109,224,559 (24). Ethiopia is divided into nine geographical regions [Afar, Amhara, Benishangul-Gumuz, Gambela, Harari, Oromia, Somali, Southern Nations, Nationalities, and People's Region (SNNPR), and Tigray] and two administrative cities (Addis Ababa and Dire Dawa). The majority of the Ethiopian population is an agrarian society, and about 83.6% live in rural areas. Agriculture accounts for approximately 43% of the country's Gross Domestic Product (GDP), and over 80% of the total population resides in the regional states of Amhara, Oromia, and SNNP (25).

In Ethiopia, the majority of the population (83.6%) lives in rural areas, and the average household size is 4.7 persons. The population is predominantly young, with more than one-third (44%) being under 15 years old, over half (52%) being between 15 and 65 years old, and only a few (3%) of all persons being over the age of 65 years. Women of reproductive age constitute 24% of the population (26, 27).

The study utilized data from the 2016 Ethiopia Demographic and Health Survey (EDHS), which is a nationally representative survey conducted every five years. Data collection was organized by the Central Statistical Agency (CSA) of Ethiopia (22) and conducted from January 18, 2016, to June 27, 2016. The study participants were women of reproductive age (15–49) who reside permanently in the selected households or stayed the night before the survey in the household (22).

### Study design, data source, and sampling procedures

A cross-sectional survey was conducted in eleven administrative regions of Ethiopia using data from the 2016 EDHS datasets, which were led by the Ethiopian Central Statistical Agency (CSA). The EDHS aims to provide valuable information on key demographic and health indicators such as fertility, family planning, infant and child mortality, maternal and child health, and nutrition in the national and sub-national areas of Ethiopia. To date, four rounds of the EDHS have been collected using similar procedures.

The survey utilized a two-stage stratified cluster sampling design. In this design, enumeration areas (EAs) or clusters (defined geographical units) were used as the primary units of data collection. These EAs were stratified based region and urban-rural residency. Each region was divided into urban and rural areas to ensure proper stratification. A total of twenty-one (21) sampling strata were created, representing the urban and rural areas in each of the nine regions and two city administrations. In the first stage of sampling, 645 EAs were randomly chosen from these strata: 202 from urban areas and 443 from rural areas. Independent selection was utilized in each sampling stratum, based on probability proportion. In the second stage, a systematic random sampling technique was used to select 28 households per EAs/cluster, ensuring equal probability (22).

For this study, the women's dataset was used. This dataset included a total of 15,683 women aged 15–49 years old from 16,650 households within 645 clusters. After weighting the sample, 7,108 currently married women were included in the final analysis. Furthermore, potential independent variables at both the individual and community levels were extracted for further analysis.

### Measurements of variables

This study examines women's empowerment as the dependent variable measured using index-based data from two key dimensions of the 2016 EDHS: decision-making power and attitudes toward the justification of wife-beating. These dimensions were chosen based on the availability of data from the 2016 Ethiopian Demographic and Health Survey (EDHS) and alignment with existing literature. To assess decision-making power, three questions were asked about women's involvement in decisions regarding their own healthcare, household purchases, and visits to family or relatives. Women's responses were categorized based on whether they made these decisions alone or jointly with their husbands. For the justification of wife-beating, respondents were asked five questions to determine whether they believed a man was justified in beating his wife under certain circumstances, such as burning food, arguing with her husband, going out without informing him, refusing sexual intercourse, and neglecting the children.

Responses to these empowerment indicators were then categorized as either empowered (scored as 1) or unempowered (scored as 0). These scores were then summed to create a composite women's empowerment score. A sum score ranging from 0 to 7 was classified as unempowered, while a score of 8 was classified as empowered. Thus, women were considered empowered if they participated in all decision-making instances either alone or jointly with their husbands, and never justified wife-beating (28). The relevant questions regarding women's participation in household decision-making and their attitudes toward wife-beating can be found in DHS Module 9 within questions 922–932 (29).

The independent variables for this study were categorized as individual, household, and community-level factors. Individual and household-level factors included sociodemographic and economic variables. On the other hand, community-level factors consisted of place of residence, region, community poverty level, and community-level media exposure.

To measure community-level media exposure, we used the proportion of women exposed to specific media as an aggregate value. Mothers’ exposure to mass media was then categorized as no exposure, less than once a week, and greater than once a week.

### Data processing and statistical analysis

The outcome variables with important predictors were extracted from the 2016 EDHS women's dataset and editing, recoding, and analysis were done using STATA 14. Descriptive statistics such as frequencies and proportions of variables were presented using tables and narrations.

Bivariable logistic and multilevel regression models were used to analyze factors associated with women's empowerment at two levels: individual and community. To capture the impact of cluster and individual-level factors on women's empowerment, a multilevel regression model that accounts for variation at these levels was used (30). Likelihood ratio, Intra-Class Correlation (ICC), and Proportional Change in Variance (PCV) were computed to measure the variation between clusters, and model comparison was conducted using deviances. The ICC coefficient quantifies the degree of heterogeneity of women's empowerment between clusters, ICC = ϭ^2^/(ϭ^2^ + π^2^/3) (31), with ϭ^2^ indicating cluster variance. PCV measures the total variation attributed to individual and community-level factors in the multilevel model compared to the null model; $\mathrm{PCV}=\frac{\mathrm{var}\;(\mathrm{null}\;\mathrm{model})-\mathrm{var}\;(\mathrm{full}\;\mathrm{model})}{\mathrm{var}\;(\mathrm{null}\;\mathrm{model})}$.

A two-level (individual and community), multilevel multivariable logistic regression analysis was used to explore factors related to women's empowerment. Furthermore, four models were created for the analysis. The first model had no variables and was used to determine the extent of cluster variation in women's empowerment. The second model included individual-level variables, the third model included community-level variables, and the fourth model included both individual and community-level variables. The model with the lowest deviance was chosen. Variables with a *p*-value of <0.2 in the bivariate analysis for both individual and community-level factors were included in the multivariable model. An adjusted odds ratio (AOR) with a 95% confidence interval (CI) and a *p*-value of <0.05 in the multivariable model indicated a significant association between the independent variables and women's empowerment.

## Results

### Individual and community-level sociodemographic and economic characteristics

A total of 7,108 women were included in the final analysis. The mean ages of the respondents were 29.3 (SD ± 6.8) years. The majority (87.6%) were rural dwellers, 41.1% were from the Oromia region and 37.4% of the participants were Muslim. Moreover, 63.4% and 56.1% of the women had no education and were not employed, respectively. Similarly, 47.7% and 64.8% of their husbands had no education and worked in agriculture, respectively. Additionally, 88.8% of the household heads were male, 42.2% of the participants were in poor wealth status, had no access to media (65.31%), and had four or more living children (48%) (Table 1).

**Table 1:** Individual and community-level sociodemographic and economic characteristics of study participants in Ethiopia, EDHS 2016 (*n* = 7,108).

Variables	Category	Frequency (*n*)	Percent (%)
Maternal age in years	15–24	1,633	23.0
	25–34	3,627	51.0
	≥35	1,848	26.0
Age at first marriage in years	<18	4,471	62.9
	18–24	2,332	32.8
	>25	305	4.3
Residence	Urban	882	12.4
	Rural	6,226	87.6
Region	Tigray	470	6.6
	Afar	67	0.9
	Amhara	1,525	21.4
	Oromia	2,921	41.1
	Somali	254	3.6
	Benishangul-Gumuz	78	1.1
	SNNP	1,556	21.9
	Gambella	19	0.3
	Harari	16	0.2
	Addis Ababa	173	2.4
	Dire Dawa	29	0.4
Religion	Orthodox	2,652	37.3
	Muslim	2,661	37.4
	Protestant	1,577	22.2
	Others^a^	218	3.1
Sex of household head	Male	6,313	88.8
	Female	795	11.2
Women's educational status	No education	4,508	63.4
	Primary education	1,995	28.1
	Secondary education	391	5.5
	Higher	214	3.0
Husband's/partner's educational status	No education	3,388	47.6
	Primary education	2,731	38.4
	Secondary education	613	8.6
	Higher	376	5.3
Women's occupation	No work	3,985	56.1
	Professional	992	13.9
	Agricultural	1,636	23.0
	Others^b^	495	6.9
Husband's/partner's occupation	No work	804	11.31
	Professional	845	11.8
	Agricultural	4,605	64.7
	Others^b^	854	12.0
Community-level poverty	Poor	3,000	42.2
	Middle	1,488	20.9
	Rich	2,620	36.8
Household wealth quintile	Poorest	1,512	21.3
	Poorer	1,582	22.2
	Middle	1,493	21.0
	Richer	1,347	18.9
	Richest	1,174	16.52
Community media exposure	No	4,643	65.3
	<Once a week	2,409	33.9
	≥Once a week	56	0.8
Number of living children	Zero	38	0.5
	One	1,337	18.8
	Two	1,242	17.5
	Three	1,079	15.2
	Four and above	3,412	48.0

^a^ Catholic, traditional and other.

^b^ Subsistence farmers, fishers, hunters, building and related trades workers, construction, main street, and related sales and service.

### The magnitude of women's empowerment

In this study, the overall magnitude of women's empowerment was 23.7% (95% CI: 22.7–24.7). Specifically, only 30.9% of women were involved in household decision-making, either alone or jointly with their husbands. The lowest level of women's decision-making power was seen in decisions regarding visits to family or relatives, with only 17.2% of women participating. Another aspect of women's empowerment was their attitude toward wife-beating, with 32.5% of women disagreeing with all justifications for it. Among these women, the highest level of empowerment was observed in cases where husbands beat their wives for refusing sexual intercourse (59.9%) (Table 2).

**Table 2:** Magnitude of women's empowerment with their respective domain in Ethiopia, EDHS 2016 (*n* = 7,108).

Variables	Yes *n* (%)	No *n* (%)
Women's participation in household decision-making	**2,194** (**30.9)**	**4,914** (**69.1)**
Usually makes decisions about own health care	1,404 (19.7)	5,704 (80.2)
Usually makes decisions about large household purchases	1,634 (23.0)	5,474 (77.0)
Usually makes decisions about visits to family or relatives	1,224 (17.2)	5,884 (82.8)
Attitude towards wife-beating	**2,311** (**32.5)**	**4,797** (**67.5)**
A husband is justified in hitting/beating their wife when she burns the food	3,934 (55.3)	3,174 (44.7)
A husband is justified in hitting/beating their wife when she argues with him	3,718 (52.3)	3,390 (47.7)
A husband is justified in hitting/beating their wife when she goes out without telling him	3,659 (51.5)	3,449 (48.5)
A husband is justified in hitting/beating their wife when she refuses to have sexual intercourse	4,260 (59.9)	2,848 (40.1)
A husband is justified in hitting/beating their wife when she neglects the children.	3,424 (48.2)	3,684 (51.8)
Overall women empowerment	**1,685** (**23.7)**	**5,423** (**76.3)**

Bold values indicate the overall magnitude of women's empowerment and the two domains.

The overall magnitude of women's empowerment varied significantly across different regions of the country. The lowest level of women's empowerment was observed in the Afar region (16.6%), while the highest was seen in Addis Ababa (69.3%) (Table 3).

**Table 3:** The magnitude of women's empowerment across regions in Ethiopia, EDHS, 2016.

Name of regions	Women empowerment			
	Yes	No	Total	Percentage
Tigray	159	533	692	23.0
Afar	104	523	627	16.6
Amhara	204	612	816	25.0
Oromia	189	764	953	19.8
Somali	278	430	708	39.3
Benishangul-Gumuz	180	403	583	30.9
SNNP	192	688	880	21.8
Gambella	144	371	515	28.0
Harari	208	209	417	49.9
Addis Ababa	339	151	490	69.3
Dire Dawa	147	280	427	34.4

### Individual and community-level determinants for women empowerment

#### Random effect analysis

The ICC in the empty model indicated that 24.4% of the total variability of women empowerment was due to cluster differences, while the remaining unexplained 75.6% was attributable to individual differences. Fifty-three percent of the variability in women's empowerment was explained by the full model and deviance was used for model comparison. As a result, the final model was the best-fitted model since it had the lowest deviance (Table 4).

**Table 4:** A random intercept model (variations) for women empowerment at cluster level by multilevel logistic regression analysis, EDHS 2016.

Measure of vibrations	Model 0 (null model)	Model 1	Model 2	Model 3 (full model)
Variance	1.0	0.6	0.5	0.5
Explained variation (PCV) (%)	Ref.	42.2	47.1	53.0
ICC (%)	24.4	16.5	34.1	48.2
Model fitness				
Log-likelihood	−3,705.6	−3,580.8	−3,599.6	−3,547.2
Deviance	7,411.2	7,161.6	7,199.3	7,094.5

Model 0: without independent variables (null model), Model 1: only individual-level variables, Model 2: only community-level variables, Model 3: individual and community-level variables (full model).

#### Fixed effects analysis

A multilevel mixed-effects binary logistic regression analysis was conducted to identify potential individual and community-level determinants of women's empowerment. In the final model (model 3), individual-level factors such as women's educational status and religion, as well as community-level variables including place of residence, region, and community wealth status, were found to be significantly associated with women's empowerment.

Accordingly, Women who attended secondary and higher education were 2.7 times (AOR: 2.7, 95% CI: 1.8–4.2) and 3.6 times (AOR: 3.6, 95% CI: 1.8–7.3) more likely to be empowered compared to those who were not educated, respectively. Additionally, Muslim women were 37% less likely to be empowered compared to Orthodox Christian women (AOR: 0.6, 95% CI: 0.5–0.8).

Women living in rural areas were 51% less likely to be empowered compared to their urban counterparts (AOR: 0.49, 95% CI: 0.3–0.8). The odds of women's empowerment in the Afar, Amhara, Oromia, SNNPR, and Gambella regions were lower by 65% (AOR: 0.35, 95% CI: 0.2–0.7), 55% (AOR: 0.45, 95% CI: 0.3–0.8), 57% (AOR: 0.43, 95% CI: 0.3–0.7), 58% (AOR: 0.42, 95% CI: 0.2–0.7), 64% (AOR: 0.36, 95% CI: 0.2–0.6), and 68% (AOR: 0.32, 95% CI: 0.2–0.5), respectively, compared to women living in Addis Ababa. Moreover, women who live in wealthy communities had a 1.6 times higher chance of empowerment compared to women from poor communities (AOR: 1.6, 95% CI: 1.1–2.4) (Table 5).

**Table 5:** Multilevel logistic regression analysis of individual and community-level factors associated with women's empowerment in Ethiopia, EDHS 2016 (*n* = 7,108).

Variables	Women's empowered		COR (95%CI)	Model 0 (ICC: 24.4%)	Model 1 AOR (95% CI)	Model 2 AOR (95%CI)	Model 3 AOR (95%CI)
	Yes *n* (%)	No *n* (%)					
Individual-level characteristics							
Mothers educational status							
No education	846 (18.8)	3,662 (81.2)	1		1		1
Primary	506 (25.4)	1,488 (74.6)	1.4 (1.2–1.8)		1.3 (1.0–1.7)		1.3 (1.0–1.7)
Secondary	197 (50.1)	196 (49.9)	3.9 (2.7–5.7)		2.9 (1.9–4.4)		**2.7** **(1.8–4.2)**^a^
Higher	136 (64.0)	77 (36.0)	7.3 (4.5–11.7)		4.6 (2.4–8.7)		**3.7** **(1.8–7.3)**^a^
Husband educational status							
No education	635 (18.8)	2,753 (81.2)	1		1		1
Primary	639 (23.4)	2,092 (76.6)	1.3 (1.0–1.6)		1.2 (0.9–1.5)		1.2 (0.9–1.5)
Secondary	221(36.0)	393 (64.0)	1.9 (1.4–2.6)		1.0 (0.7–1.5)		0.9 (0.7–1.4)
Higher	191 (50.9)	184 (49.0)	3.8 (2.6–5.5)		1.2 (0.7–1.9)		1.10 (0.7–1.8)
Sex of household head							
Male	1,445 (22.9)	4,868 (77.1)	1		1		1
Female	240 (30.3)	555 (69.7)	1.4 (1.1–1.8)		1.3 (1.0–1.7)		1.2 (0.9–1.6)
Women's occupation							
No work	908 (23.4)	2,972 (76.6)	1		1		1
Professional	239 (28.3)	605 (17.7)	1.1 (0.8–1.5)		0.9 (0.6–1.5)		0.9 (0.6–1.5)
Agricultural	297 (32.2)	1,338 (81.8)	0.7 (0.6–0.9)		0.8 (0.6–1.1)		0.5 (0.6–1.2)
Others^a^	241 (23.7)	508 (67.8)	1.4 (1.1–1.9)		0.9 (0.6–1.4)		0.9 (0.6–1.4)
Religion							
Orthodox	769 (29.0)	1,885 (71.0)	1		1		1
Muslim	5,319 (20.0)	2,130 (80.0)	0.6 (0.4–0.8)		0.7 (0.5–0.9)		**0.6** **(0.5–0.8)**^a^
Protestant	1,218 (77.3)	359 (22.7)	0.8 (0.6–1.1)		0.8 (0.6–0.9)		0.8 (0.5–1.1)
Other**	28 (12.8)	190 (87.2)	0.6 (0.2–1.7)		0.7 (0.2–1.7)		0.6 (0.2–1.7)
Women's employment status							
Unemployed	1,152 (22.4)	3,985 (77.6)	1		1		1
Employed	533 (27.0)	1,438 (72.9)	1.1 (0.9–1.4)		0.9 (0.7–1.4)		0.9 (0.7–1.3)
Household wealth status							
Poorest	299 (19.8)	1,212 (80.2)	1		1		1
Poorer	265 (16.8)	1,318 (83.3)	0.8 (0.6–1.1)		0.8 (0.6–1.0)		0.8 (0.5–1.1)
Middle	287 (19.2)	1,206 (80.8)	0.9 (0.7–1.3)		0.9 (0.6–1.2)		0.7 (0.4–1.1)
Rich	338 (25.1)	1,009 (74.9)	1.3 (0.9–1.8)		1.1 (0.77–1.5)		0.8 (0.4–1.1)
Richest	497 (42.3)	677 (57.7)	2.7 (1.9–3.7)		1.5 (1.1–2.28)		0.8 (0.4–1.5)
Age at first marriage in years							
Less than 18	983 (22.0)	3,487 (78.0)	1		1		1
18–24	601 (25.7)	1,732 (74.3)	1.1 (0.9–1.4)		0.9 (0.7–1.1)		0.9 (0.7–1.1)
25+	102 (33.5)	203 (66.6)	1.5 (1.1–2.3)		1.1 (0.7–1.7)		1.0 (0.6–1.6)
Community-level characteristics							
Residence							
Urban	426 (48.3)	456 (51.7)	1			1	1
Rural	1,259 (20.2)	4,967 (79.8)	0.2 (0.16–0.3)			0.3 (0.2–0.4)	**0.5** **(0.3–0.8)**^a^
Region							
Addis Ababa	119 (69.3)	53 (30.7)	1			1	1
Afar	11 (16.6)	56 (83.4)	0.1 (0.03–0.1)			0.3 (0.1–0.5)	**0.4** **(0.2–0.7)**^a^
Amhara	382 (25.0)	1,143 (74.9)	0.1 (0.07–0.2)			0.4 (0.3–0.7)	**0.5** **(0.3–0.8)**^a^
Oromia	578 (19.8)	2,343 (80.2)	0.1 (0.05–0.1)			0.3 (0.2–0.5)	**0.4** **(0.3–0.7)**^a^
Somali	100 (39.3)	154 (60.7)	0.2 (0.16–0.4)			1.1 (0.6–1.9)	1.5 (0.8–2.7)
Benishangul	24 (30.9)	53 (69.1)	0.2 (0.09–0.3)			0.7 (0.4–1.2)	0.8 (0.4–1.5)
SNNP	339 (21.8)	1,216 (78.2)	0.1 (0.06–0.2)			0.4 (0.2–0.6)	**0.4** **(0.24–0.7)**^a^
Gambella	5 (27.9)	13 (72.1)	0.1 (0.08–0.2)			0.4 (0.2–0.7)	**0.4** **(0.2–0.7)**^a^
Harari	7 (46.9)	8 (53.0)	0.4 (0.25–0.8)			1.0 (0.6–1.7)	1.4 (0.8–2.3)
Dire Dawa	10 (34.4)	19 (65.6)	0.2 (0.11–0.4)			0.4 (0.2–0.8)	0.6 (0.3–1.1)
Tigray	108 (22.9)	362 (77.0)	0.1 (0.06–0.2)			0.4 (0.2–0.6)	**0.3** **(0.2–0.6)**^a^
Community poverty status							
Poor	598 (20.0)	2,402 (80.0)	1			1	1
Middle	330 (22.2)	1,158 (77.9)	1.2 (0.9–1.5)			1.2 (0.9–1.5)	1.3 (0.9–1.8)
Rich	758 (29.0)	1,862 (71.0)	1.6 (1.3–2.1)			1.6 (1.3–2.1)	**1.6** **(1.1–2.4)**^a^
Community media exposure							
No exposure	3,698 (79.7)	944 (20.3)	1			1	1
<Once a week	704 (29.2)	1,706 (70.8)	1.1 (1.2–1.8)			1.1 (0.8–1.3)	0.9 (0.7–1.2)
≥Once a week	37 (66.9)	19 (33.0)	5.4 (2.3–2.4)			2.4 (1.1–5.6)	1.6 (0.7–3.4)

** Catholic and traditional religions followers.

^a^ (in bold) Statistically significant at *p*-value <0.05 at model 3; Model 0, a model for the intra-class correlation coefficient (null model), Model 1, Individual-level characteristics, Model 2, Community-level characteristics, Model 3, Both individual and community-level characteristics (full model).

## Discussion

The overall magnitude of women's empowerment in Ethiopia was found to be 23.7% (95% CI: 22.7–24.7), with 30.9% of women participating in household decision-making. Specifically, only 19.75% of women were involved in decisions regarding their healthcare. This finding was unexpectedly lower than a previous study using EDHS 2005 data, which showed that 29.3% of women had a say in their healthcare needs (32). The possible reason for the discrepancy might be due to the donor-driven nature of maternal and child healthcare programs, which may lack sustainability after the termination of funding, the reduced functionality of women's associations, and the poor involvement of husbands in maternal and child health decisions. Furthermore, it is possible that in 2016, women were more willing to express the limitations they faced in decision-making due to increased awareness of gender issues. This awareness could have led to more honest reporting compared to 2005, when they might not have openly acknowledged these constraints.

However, our results were significantly lower than a study in Bangladesh, where nearly 45% of women participated in healthcare decisions, large household purchases, and family visits (33). Similarly, our findings were lower than those in Pakistan and India. In Pakistan, 36.6% of women were involved in household decision-making (34), while in North India, 53% participated in major household decisions (35). In Nepal, 72% of women were involved in decisions regarding household purchases (36).

Moreover, our finding is also slightly lower compared to other African countries. For instance, approximately 37% of women participated in at least one household decision in Mali (37) and 35.9% in the Democratic Republic of the Congo (DRC) (38). The possible reason for the discrepancy might be measurement and study setting differences, in this study women's participation in decision-making was measured by considering the composite value of decision-making indicators like decision-making on health care, household purchases, and a family visit whereas empowerment in India was measured in terms of women's autonomy, which is slightly different. Additionally, the study settings might contribute to discrepancies. Gender equality and women's participation in many aspects are higher in developing countries like Bangladesh, Nepal, and Pakistan compared to Ethiopia. However, in Ethiopia, women's participation remains a significant public health policy concern.

This study showed that educational status significantly impacts women's empowerment, with respondents who had completed primary education or higher being more empowered than those with no formal education. This result aligns with studies conducted in southern Ethiopia (39) and other African countries, where higher educational attainment is linked to increased empowerment for women (40–44). Similarly, studies from different Asian countries highlight education as a significant factor in positively influencing women's empowerment (33, 34, 41, 45–51). This might be due to the fact that education increases individuals’ awareness and enhances self-esteem. When women become more educated, they become more informed about their rights and are more likely to advocate for and uphold these rights (52). This finding is also supported by the Human Capabilities Theory by Martha Nussbaum and Amartya Sen, which emphasizes the role of education in expanding individual capabilities, fostering autonomy, and promoting overall empowerment and well-being (53, 54).

Besides, the findings of this study revealed a significant association between residency and women's empowerment. Respondents from rural areas were less empowered compared to those from urban settings. This finding is consistent with studies from various African and Asian countries, indicating that women in urban areas experience higher levels of empowerment than their rural counterparts (17, 44, 55, 56). This disparity may be attributed to limited access to information on women's rights and autonomy among women living in rural settings. Additionally, awareness levels within rural communities regarding women's rights and autonomy tend to be lower compared to urban areas.

Participants who identify as Muslim exhibit lower levels of empowerment compared to their Christian counterparts. Research conducted in Africa on the impact of religion on the Millennium Development Goals has similarly shown that being Muslim is associated with lower female school participation rates, less non-agricultural employment among women, and lower representation of women in government (57). Other studies also highlighted that gender inequality tends to be more pronounced among Muslims and Hindus compared to Christians and Buddhists (58, 59). This disparity may stem from cultural challenges in reconciling religious values with traditional beliefs and practices. Gender inequality often persists due to cultural norms and interpretations of religious teachings, which are prevalent in Muslim societies.

The level of community poverty significantly influences women's empowerment. Women residing in the wealthy communities exhibit higher levels of empowerment compared to those in poorer communities. It is well known that poverty constrains women's self-determination and limits their participation in economic, social, and political spheres. It is widely recognized that poverty is a fundamental driver of gender inequality, which hinders women's economic and social empowerment (60, 61). Moreover, women and girls living in poverty are more vulnerable to sexual exploitation due to their lack of income and resources (62–64).

In this study, the geographical region where women reside was found to significantly impact their empowerment. Women living in Afar, Amhara, Oromia, SNNPR, Gambella, and Tigray regions exhibited lower levels of empowerment compared to those residing in Addis Ababa city administration. This disparity can be attributed to the greater investment by the government and non-governmental organizations in Addis Ababa in areas such as education, decision-making and economic empowerment initiatives for women. Additionally, differences in socio-economic status between urban and rural settings may also contribute to variations in women's empowerment levels.

In this study, variables such as occupation and community media exposure, previously identified as key contributors to women's empowerment (65–67), did not show statistically significant associations with empowerment. This contrast may stem from contextual differences between study populations, as the impact of these factors can be highly context-dependent. Additionally, variations in the definition or measurement of empowerment might account for the discrepancy. Furthermore, unmeasured variables such as cultural norms or regional policies may have played a more dominant role, minimizing the influence of occupation and media exposure.

### Strengths and limitations

The study utilizes nationally representative datasets, enhancing the generalizability of its findings to married women of reproductive age across Ethiopia. Besides, employing a multilevel modelling technique takes into consideration the hierarchical structure of the survey data, thereby ensuring more robust and valid results. However, a limitation of the study is the absence of qualitative methods, which could have been valuable in exploring the attitudes and beliefs of women in greater depth.

## Conclusion

This study reveals a low overall magnitude of women's empowerment. Maternal education level and religion appeared as significant individual-level factors, while community wealth status, place of residence, and region were notable community-level variables associated with women's empowerment. To address these findings, the Ethiopian government needs to prioritize strategies that enhance maternal education and increase women's involvement in household decision-making, thereby promoting empowerment. Additionally, implementing community-based empowerment programs, including enhancing women's access to media and health information, and involving them in income-generating activities, could serve as effective interventions to empower women.

## Data Availability

The original contributions presented in the study are included in the article/Supplementary Material, further inquiries can be directed to the corresponding author.
